# Evaluation of optimal strategies for breast cancer screening in Ghana: A simulation study based on a continuous tumor growth model

**DOI:** 10.1371/journal.pone.0323485

**Published:** 2025-06-17

**Authors:** Asamoah Larbi, Eric Nyarko, Samuel Iddi

**Affiliations:** 1 Department of Statistics and Actuarial Science, School of Physical and Mathematical Sciences, University of Ghana, Legon, Accra, Ghana; 2 African Population and Health Research Center, APHRC Campus, Manga Close, Off Kirawa Road, Nairobi, Kenya; Local Health Authority Caserta: Azienda Sanitaria Locale Caserta, ITALY

## Abstract

Mammographic breast cancer screening plays a crucial role in detecting small tumors, which can prevent the progression of the disease and reduce the risk of breast cancer mortality. This study aimed to evaluate optimal strategies for a breast cancer screening program in Ghana. A continuous growth model was employed to evaluate the natural history of breast cancer in Ghana, from its onset to detection. We estimated tumor growth rates and the age at which symptomatic detection occurs using the maximum likelihood estimation method based on clinical data from the National Center of Radiotherapy and Nuclear Medicine at Korle Bu Teaching Hospital. Our results revealed that biennial screening provided a better trade-off between interval cancers and overdiagnosis than annual or triennial intervals. The simulation results for early screening under biennial intervals showed an average detection age of 47 years for unscreened individuals (control group) and 46 years for those screened (intervention group). While the screening approach (50–69 years) with biennial screening proved more reliable than other strategies, the early screening approach (30-65 years with biennial screenings) provided certain advantages in detection for the Ghanaian population. Our findings highlight the importance of early detection and advocate for the systematic adoption of mammography in Ghana and other low- and middle-income countries, contributing to enhanced breast cancer screening and patient treatment plans, as well as informing policy development.

## Introduction

Breast cancer is a significant health issue for women. In 2022, approximately 2.3 million women worldwide were diagnosed with breast cancer, and the disease resulted in 670,000 deaths [1]. Most of these cases are presumably early-stage tumors in high-income regions of America and Europe, mainly due to the effective detection of small tumors through organized and non-organized screening programs. Population-based screening initiatives enable healthy women to participate in regular mammography screenings according to a defined schedule. These screening programs involve multi-stage processes aimed at identifying individuals who may have the disease through a series of scheduled examinations [2]. The primary goal is to recognize individuals at risk of breast cancer and to provide early intervention options, which may help reduce the incidence of secondary breast cancer cases and associated deaths [2].

Evidence suggests that active screening programs can greatly reduce the incidence of late-stage breast cancer and decrease mortality rates [3]. In countries where these programs are well-established, mammographic screenings have resulted in a 30% decrease in advanced tumors, lymph node involvement, and breast cancer deaths [4].

Despite the high mortality rates associated with breast cancer in Sub-Saharan Africa (SSA), mainly due to the absence of screening programs and awareness campaigns, late detection remains a critical issue [3]. For instance, Ghana lacks a dedicated screening program, resulting in an increase in late-stage breast cancer cases and corresponding high mortality rates [5]. A study conducted in 97% of hospitals in the Eastern region revealed a breast cancer prevalence of 4.5% among adult women [6]. In 2012, a review of patients at Korle Bu Teaching Hospital indicated that out of 1,136 cancer patients, 333 were diagnosed with breast cancer, representing 29.3% of all cases and a prevalence rate of 80.5 cases per 100,000 people [7]. Unfortunately, many of these cases are diagnosed at advanced stages, reducing survival rates.

Therefore, early detection and treatment of breast cancer are crucial in low—and middle-income countries (LMICs) like Ghana. One primary intervention is the introduction of intensive, population-based mammography screening programs. Although many developing countries lack the necessary resources, these screening programs are essential to prevent late-stage diagnoses and improve quality of life. For example, Ghana could adopt a systematic approach to breast cancer screening that begins with opportunistic screening, transitions to organized and centralized screening, and eventually establishes a formal screening program as the country’s healthcare resources improve significantly.

Various countries and international organizations recommend established evidence-based screening strategies. The main consideration in adopting organized or population-based screening programs is determining which strategy or policy to prefer. Understanding country-specific evaluations of screening strategies is valuable, as it allows for a focused discussion on the evaluation relevant to the population in question.

In Sweden, the continuous growth models have primarily been applied to its screening program, which targets individuals aged 40 to 70 and follows a biennial screening frequency [8]. This strategy was chosen to effectively apply the model to our data, aligned with the standard recommendations from the International Agency for Research on Cancer (IARC), which suggest screening for those aged 50 to 69 years every two years [9]. To ensure the relevance of our findings to the Ghanaian population, we calibrated the sensitivity parameters using growth rate estimates derived from empirical data, conducting sensitivity analysis under various scenarios. Given the genetic differences between the two regions, confirming that the sensitivity values adopted from prior studies were suitable for our current research was essential. We aim to evaluate optimal screening strategies for Ghana, contributing to key recommendations, particularly those from the IARC. We strive to provide geographic-specific analysis and assumptions, particularly on Ghana. Ultimately, we aspire to inform the development of an organized or centralized screening program in the future and guide research that involves more representative data to shape national screening program policies for LMICs like Ghana.

## Breast cancer screening program

Breast cancer screening programs can be categorized as either population-based or opportunistic. Population-based screening aims to identify early signs of the disease in healthy individuals, with screenings typically taking place every 18 to 24 months [10]. In contrast, opportunistic screening is less expensive and can occur whenever a woman visits a healthcare facility [11]. The benefits of mammography screening include a reduction in late-stage breast cancer cases, prevention of disease progression, and lower mortality rates [2,12]. However, there are potential drawbacks, such as overdiagnosis, false positives, false negatives, and interval cancers. Overdiagnosis can lead to unnecessary treatments, while false positives may cause significant anxiety for patients [13]. Interval cancers, detected between screenings, are particularly concerning as they often involve high-grade tumors and increase the risk of death [14]. It is crucial to weigh both the benefits and harms of screening programs to ensure effective breast cancer care.

The balance between the benefits and harms of screening varies based on how frequently screenings are conducted [15,16]. For instance, the World Health Organization (WHO) recommends mammography screening programs based on age and resource availability. In well-resourced settings, WHO strongly advises organized mammography screening for women aged 50–69 every two years. This recommendation is supported by significant reductions in mortality observed in high-income countries implementing such programs due to early detection, effective diagnosis, and treatment. In contrast, evidence supporting mammography screening in limited-resource settings is lacking and heavily dependent on the healthcare system’s capabilities. For women aged 40–49 and 70–75, WHO provides a conditional recommendation based on limited evidence, which stems from lower incidence rates and reduced mammography sensitivity. Furthermore, in resource-limited settings, WHO advises against screening programs for these age groups unless moderate-quality evidence supports their implementation [16]. Moreover, the IARC evaluated breast cancer screening and found sufficient evidence that mammography can reduce mortality among women aged 50–59 [9]. However, the evidence for women aged 40–49 is limited, while there is inadequate evidence for women under 40 and those over 69. Consequently, IARC recommends breast cancer screening programs explicitly targeting women aged 50–69. In 2014, a study [17] included in the IARC handbook expanded the eligible population for screening to women aged 50–74 based on sufficient evidence that mammography reduces breast cancer-related deaths in this group. However, significant risks of overdiagnosis were also noted in these cases.

It is worth noting that different high-income countries have established screening programs guided by evidence-based recommendations. In the United States, it is recommended that high-risk women aged 50–74 undergo annual mammography, and this recommendation has been extended to include women aged 40–49 [18]. Conversely, in Canada, routine screening for this age group is not recommended due to the low risk of cancer [19]. Japan and Brazil recommend screening for women aged 40–74, with additional imaging for those at high risk [20]. Screening guidelines can differ by region based on age, risk factors, and available screening technologies. In Africa, there needs to be more evidence-based recommendations explicitly tailored to their population. Most existing guidelines are derived from high-income countries, highlighting the need for tailor recommendations for LMICs like Ghana. This is essential to address the unique contextual needs of the populations. Given the moderate evidence and the improvements in the healthcare system mentioned in the WHO’s recommendations, Ghana stands to benefit from screening, especially early screening [21]. This study used a simulation-based continuous growth model to address the insufficient evidence and the lack of studies supporting early screening programs, as highlighted in the IARC Handbook and WHO recommendations.

## Natural history through the lens of quantitative models

One key advantage of screening programs is their ability to provide a clear understanding of the natural history of breast cancer, from the condition’s onset to its detection [2]. This understanding is crucial for developing effective treatment strategies and improving patient outcomes. It underscores the significance of the audience’s research and its potential impact on patient care.

Research utilizing mathematical and statistical models has proven essential in studying breast cancer. One commonly used approach is the Multi-state Markov model (MSM), which helps researchers gain insights into the natural history of the disease [22]. These models are beneficial for analyzing tumor growth and evaluating the effectiveness of screening programs, with both three-state and five-state models being prevalent choices. The three-state model categorizes the natural history of breast cancer into three primary stages: the tumor latency stage, the detectable tumor stage, and the tumor progression stage [22]. In contrast, the five-state model provides a more detailed breakdown of these stages into five distinct phases, namely disease-free, pre-clinical (node negative), pre-clinical (node positive), clinical (node negative), and clinical (node positive) [23]. However, concerns regarding the biological interpretation and pragmatic assumptions underlying these models have declined their application [8].

In recent years, Continuous Growth Models (CGMs) have emerged as more flexible alternatives, offering a biologically based perspective on estimating tumor growth rates. CGMs are increasingly employed in breast cancer research to enhance understanding of the disease’s natural history and to inform screening program criteria. These models use various growth functions, such as exponential growth [24], logistic equations, and hazard functions, to analyze changes in population size and growth rates. For example, the logistic equation assesses changes in population size relative to the current population [24,25], while the hazard function evaluates the likelihood of population decline or death risk based on factors like age at onset and growth rate [26]. Previous studies applying CGMs employed various quantitative assumptions to characterize different stages of breast cancer’s natural history or sub-models, including detection modes as explored in [8,27].

## Continuous growth models

### Tumor onset

The onset of breast cancer tumors has been estimated based on age [8]. Tumor onset is defined as the formation of the first malignant cell. The age at onset has been modeled using the Moolgavker-Venzon-Knudson (MVK) two-state carcinogenesis model [8]. In this model, the age at which the tumor is considered to have formed is when it reaches 0.5 mm in diameter. However, this assumption differs from the actual size of a single malignant cell, which ranges from 0.01 mm to 0.02 mm. The MVK assumption was modified to facilitate the interpretation of parameters, as histological examination typically reports tumor sizes in increments of 0.5 mm. In a similar study, a biologically based two-state clonal expression (TSCE) model was used to analyze tumor onset for lung cancer. This model incorporated hazard rate and survival probability functions [28].

In this study, breast cancer incidence is determined as a function of age using MVK carcinogenesis assumptions as adopted by [8] with a probability density function given as

$$\;f_{T}(t)=-\frac{dG_{T}(t)}{dt}=\delta AB(B-A{)}^{\delta }\frac{e^{\delta Bt}(1-e^{(B-A)t})}{(Be^{(B-A)t}-A{)}^{\delta +1}},$$ (1)

where *G*_*T*_(*t*) is the survival function (see Eq (2), and *A*, *B*, and δ are the model parameters, which are functions (see Eq (3)–Eq (5)) of the four Poisson events rate: cell division ($\tilde{\alpha }$), first mutation ($\tilde{\nu }$), cell death ($\tilde{\beta }$), second mutation ($\tilde{\mu }$) [8], *t* is the time or age at which cancer onset risk was evaluated. However, since the onset estimates [29] were based on Northern European incidence rates, we anticipated that the onset risk estimates for Ghana (West Africa) would be approximately 41.5/86.4 = 0.48 times lower, according to the GLOBOCAN 2020 statistics for age-standardized incidence rates [5].

### Tumor growth

Breast cancer tumor growth has been described mathematically in previous research [30]. This model assumes that tumor size depends on the rate of cell reproduction, following either an exponential growth curve or an inverse growth rate after the onset of cancer. In its simplest form, tumor volume is expressed as a function of the volume of a single cell. Due to variations in growth rates among individuals, the inverse growth rate is modeled as a random variable that follows a gamma distribution. Additionally, tumors are assumed to be spherical, with tumor size further expressed in either tumor volume or diameter. Isheden and Humphreys [27] also adopted the growth model used by [30]. However, the study specifically defined the volume of a single cell as a tumor with a diameter of 0.5 mm. Abrahamsson [30] also assumed that tumor growth is spherical and follows an exponential growth curve with constant doubling time. [29] also presumed that tumor growth followed exponential growth for a given age at onset *T = t* , and inverse growth rate *r*, and the time constant of the growth rate was explained using the doubling time of the tumor (i.e., DoublingTime=ln(2)r); single cell volume $\nu_{o}$, was assumed to be spherical with diameter 0.5 mm (i.e., v0 was approximately $0.065\;{\text{mm}}_{3}$). [27].

Consequently, tumor volume at age *x* (x≥t), *V*(*x*), was expressed as

$$V(x)=\nu_{o}e^{\frac{x-t}{r}},\;x>t.$$ (2)

Given that the inverse growth rate *r*, can be modeled as a random variable (*R*) from a gamma distribution with parameters *a* and *b* (for *a* = *b*), we were able to account for heterogeneity in individual tumor growth as

$$f_{R}(r)=\frac{b^{a}}{\Gamma (a)}r^{a-1}e^{-br},\;a,b,r>0.$$ (3)

### Symptomatic detection

Abrahamsson [30] estimated the time from the onset of malignancy to the point of symptomatic detection, highlighting that this duration varies according to the tumor and adheres to a hazard function. Similarly, Isheden and Humphreys [27] proposed that the probability of symptomatic detection at a given time is influenced by the age at which the disease begins and is also a function of tumor size as determined by the hazard function. Strandberg and Humphreys [8] further suggested that the time from tumor onset to symptomatic detection follows a continuous hazard rate function proportional to latent tumor volume, which correlates with tumor size according to the hazard function. Moreover, Abrahamsson *et al*. [31] proposed that the time to symptomatic detection also depends on the hazard function.

Assuming the random variable U = T + U′, where *U* denotes age at symptomatic detection, and U′ is the time from tumor onset to symptomatic detection, then we can express the rate at which symptoms are noticed by an individual based on Strandberg and Humphreys’s [8] assumptions as

$$P{(}^{U^{\prime }}\in (x,x+\Delta x]{|}^{U^{\prime }}>x)=\eta V(x)\Delta x+o(\Delta x),\;\eta >0$$ (4)

where η measures hazard rate, and *V*(*x*) in the tumor volume.

### Screen detection (screening sensitivity)

Abrahamsson [30] predicted the sensitivity of mammography screening using a logistic function to calculate the probability of detecting breast cancer tumors based on a given percentage of mammographic density. They assumed that the logistic model would improve the fit of their predictions. Similarly, Strandberg and Humphreys [8] proposed that the probability of detecting an existing tumor during a screening program could also be modeled using a logistic function. Based on the assumptions put forth by Strandberg and Humphreys regarding screening sensitivity, the probability of detecting an existing tumor at the screening age *x* can be expressed as

$$\frac{e^{\beta_{o}+\beta_{1}d(x)}}{1+e^{\beta_{o}+\beta_{1}d(x)}},\;x>T,$$ (5)

where $\beta_{0}$ and $\beta_{1}$ are sensitivity parameters, *d*(*x*) is the tumor diameter at age (*x*). It is important to note that we calculated screening sensitivity using the model described above in this study. The parameters $\beta_{0}$ and $\beta_{1}$ were obtained from [32], as there was a lack of screening data in Ghana. We calibrated the model using our growth rate parameters to minimize potential uncertainties to fit the Ghanaian screening population. We also conducted a sensitivity analysis by varying the parameters by ±10% to support our assumptions.

## Estimation of model parameters

Previous studies have employed maximum likelihood estimation to derive parameter values effectively. For example, Abrahamsson [30] utilized two distinct maximum likelihood functions to estimate unknown parameters in their model jointly. Strandberg and Humphreys [8] also jointly estimated the age at onset and tumor size by applying a maximum likelihood function. Moreover, Abrahamsson *et al*. [31] estimated their model parameters by optimizing a likelihood function. In our study, we adopted the model properties described by Plevritis *et al*. [33] to facilitate our parameter estimation, following the approaches described by Isheden *et al*. [34] and Syriopoulou *et al*. [35]. Specifically, we focused on the distribution of tumor volume at the time of symptomatic detection, denoted as VU = ν, conditioned on a fixed inverse growth rate *R* = *r*, as demonstrated below (see [36] for the derivation).

$$f_{V_{U}}(V_{U}=\nu |R=r)=\eta r\;exp(-\eta r(\nu -\nu_{o})),\;\nu >\nu_{o}$$ (6)

where η is the risk of symptomatic detection, and $\nu_{0}$ is tumor volume at onset. Secondly, the joint density function for tumor volume at symptomatic detection as

$$f_{V_{U},R}(\nu ,r)=\eta \frac{b^{a}}{\Gamma (a)}r^{(a+1)-1}\;exp(-r(b+\eta (\nu -\nu_{o}))),\;\nu >\nu_{o}$$ (7)

where *a* and *b* are the shape and scale parameters of the gamma growth rate, *r*, respectively. Third, the marginal distribution of tumor volume at symptomatic detection $V_{U}$, conditioned on a fixed growth rate *R* = *r*, is expressed as

$$f_{V_{U}}(\nu )=\eta a\frac{b^{a}}{(b+\eta (\nu -\nu_{o}){)}^{a+1}},\;\nu >\nu_{o}.$$ (8)

Given the marginal distribution for tumor volume at symptomatic detection $f_{V_{U}(\nu )}$ above, we used the maximum likelihood principle to obtain the estimated values $\hat{\theta }=(\hat{\eta },\hat{a},\hat{b})$, based on the likelihood function *L* and the log-likelihood function *l*, under the assumption that the true parameter is θ=(η,a,b) such that the maximum likelihood estimator, ${\hat{\theta }}_{MLE}$ is the value of θ=(η,a,b) that maximizes L(θ), ${\hat{\theta }}_{MLE}=\arg\max_{\theta }L(\theta )$ [37]. (See Eq (6) - Eq (9)) for the derivation of the log-likelihood function.

$$L(\theta )=\prod_{i=1}^{n}f_{V_{U}}(\nu_{i})$$ (9)

l(θ)=logL(θ) (10)

$$=\sum_{i=1}^{n}\log f_{V_{U}}(\nu_{i})$$ (11)

## Data source

The study analyzed retrospective cohort data from breast cancer patients treated at Korle Bu Teaching Hospital from January 2018 to December 2022. Essential information, including tumor size, stages, and characteristics, was extracted from medical records such as mammograms and biopsy reports from 5/10/2023 to 3/11/2023. Only individuals with complete screening records were included, while those without complete information were excluded. A total of 187 cases with detailed tumor size information were used. Data were obtained from the Department of Nuclear Medicine and Radiotherapy of the National Center of Radiotherapy and Nuclear Medicine at Korle Bu Teaching Hospital and entered into Excel for analysis using R software.

## Simulation study

### Simulation parameters

Presented in Table 1 are the parameters used in the simulation study. In order to model a screening program to determine the optimal screening age range for Ghana, we needed to model risk of onset Eq (1) and screening sensitivity Eq (5), but the data available was clinical data, which lacked screening information. However, with the simulation study described in the [sec: 7.2]simulation procedure, we were able to borrow parameter estimates of onset risk (*A*, *B*, and δ) and screening sensitivity ($\beta_{0}$ and $\beta_{1}$) from previous studies [32,38]. Based on the onset assumptions on GLOBOCAN statistics for the incidence rates in Northern Europe in relation to the incident rates in West Africa [5], we were able to account for the onset of population-specific disease. Then, using maximum likelihood estimation (outlined in the [s: 5]estimation of model parameters and estimates in Table 2), we estimated tumor growth parameters (*a* = *b*) and symptomatic detection (η) using the empirical data described in the [s: 6]data source, to model tumor growth Eq (3) and symptomatic detection Eq (4). Using this approach, we were able to tailor the screening program to the Ghanaian population.

**Table 1:** Parameters used in the simulation study.

Model	Parameter	Estimate	Method of estimation
1	A,B,δ	–0.0722, $1.18\times 10^{-3}$, 0.0952	Literature
1	*t*	0.48 × t ((41.5/86.4)=0.48)	Theoretical assumption
3	a,b		Empirical data (Tumor size)
4	η		Empirical data (Tumor size)
5	$\beta_{0},\;\beta_{1}$	–5.45, –5.04, –4.67, 0.48, 0.56, 0.65	Literature

**Table 2:** Estimates of tumor growth rate and age at symptomatic parameters.

Parameter	Point estimation	Asymptotic 95% CI	Std Error
η	$6.481\times 10^{-5}$	($4.909\times 10^{-5}$, $8.054\times 10^{-5}$)	$9.561\times 10^{-6}$
*b*	$8.162\times 10^{-1}$	($7.832\times 10^{-1}$, $8.492\times 10^{-1}$)	$2.006\times 10^{-2}$
*a*	*b*	-	-

### Simulation procedure

#### Natural history model without screening

**Steps:**

1. **Simulate disease population**: We simulated 5,000 births into the disease population across 35 different cohorts (e.g., from 1985 to 2020) on a yearly basis. The simulation was based on the assumption that each individual has a certain probability of developing the disease at any given time but will only experience a single tumor in their lifetime.
2. **Simulate tumor onset age, *t***: To simulate the natural history in the absence of screening, we first simulated the age of disease onset using the probability of disease occurrence described in Eq (1), applying the following estimates: $A=-0.0722,\;B=1.18\;*\;10^{\left(-3\right)},\;\delta =0.0952,\;t=c(0,38:92)$ [29]. We then sampled time or age at onset using *sample* in "R" and setting *x* = *t*, *n*(size) = number of birth cohorts (e.g., 1985-2020), and probability = probability of disease onset.
3. **Simulate inverse growth rate, *R* = *r***: We simulated tumor growth rate *r*, from a gamma distribution Eq (3) using the shape parameter *a* = 0.816 and scale parameter 1/b(a=b), obtained from the empirical data.
4. **Simulate tumor volume at symptomatic, *V*(*U*)**: We simulated tumor volume at symptomatic Eq (21). The estimate of hazard rate η = *exp*(-9.644), tumor growth rate *r*, volume at onset $\nu_{o}$ was approximately $0.065\;{\text{mm}}_{3}$, and *u* was sampled from a uniform distribution *U*[0,1].
5. **Simulate size at symptomatic detection, *d*(*U*):** We simulated the size at symptomatic detection (diameter) by converting the volume at symptomatic detection Eq (22).
6. **Simulate age at symptomatic detection, *U***: Symptomatic detection ages were simulated by adding the age at disease onset, *T*, and time to symptomatic detection U^′^.

#### Natural history model with screening

**Steps:**

1. **Simulate natural history without screening:** We used the simulated natural history without screening above and imposed screening modalities as defined by the steps below.
2. **Simulate screening attendance probability**: We simulated the probabilities of individuals attending screenings. For perfect screening, we assumed that all individuals would attend the screening at time *t*. In the case of imperfect screening, we assumed that 80% of individuals would participate with a probability of 0.90 at time *t*. Additionally, 20% of individuals would attend with a probability of 0.15 at time *t*. There was a 0% probability of individuals not attending any screening at any time *t* (indicating no screenings).
3. **Simulate tumor volume at screen detection, *V*(*x*)**: The tumor volume at screen detection was simulated using Eq (22). The variables were age or time at detection *(x)*, the simulated age at onset, growth rate, *r*, and initial tumor volume, $\nu_{o}$, approximately $0.065\;{\text{mm}}_{3}$.
4. **Simulate the size at detection, *d***_***r*,*t***_**(*x*)**: We calculated the tumor size or diameter at screen detection by converting the volume to tumor size or diameter at the screen as expressed in Eq (24).
5. **Simulate screening sensitivity:** We simulated screening sensitivity Eq (25) for a given screening planned time, *t*, generated from an appropriate Bernoulli distributions. Screening sensitivity was calculated using the estimates $\beta_{0}$ = -5.45, -5.04, and -4.67 [32] for low, moderate, and high sensitivity scenarios, respectively, and $\beta_{1}$ = 0.48, 0.56, and 0.65 [32] for low, moderate and high sensitivity scenarios respectively [35] ; and tumor size at screen, *d*(*x*).

## Experimental assumptions

### Selection of screening sensitivity under different scenarios

Simulations were performed based on various model assumptions for screening sensitivity in Eq (5) [32]. The sensitivity of the screening program was evaluated under low to high scenarios (see simulating step for screening sensitivity) to understand the variability of screening sensitivity while considering potential overdiagnosis for women attending any of these screening programs. The optimal scenario-sensitive parameters were held fixed for the next step.

### Selection of screening ages

To determine the appropriate screening ages for the screening program in Ghana, we conducted a sensitivity analysis by varying the screening ages across three options. This analysis was based on our previous experiment’s selected screening sensitivity and attendance rates. The goal was to control the number of symptomatic tumors detected before and after the adjustments to the screening ages, thereby increasing the probability of detecting tumors through screening. We aimed to ensure that the proportion of screen-detected tumors aligned with the detection proportions observed in countries implementing population-based screening programs.

### Selection of screening frequency or interval

The study assumed three different screening strategies to obtain an optimal screening interval [39], which involved varying the screening frequency to include annual screening, biennial screening, and triennial screening for the screening ages selected from the previous experiment [40].

### Ethical considerations

The study was approved by the Scientific and Technical Review Committee of Korle Bu Teaching Hospital, and ethical approval was granted by the Institutional Review Board (IRB) of Korle Bu Teaching Hospital (Reference No.: KBTH-IRB 00058/2023). The data were fully anonymized before being accessed, and the IRB ethics committee waived the requirement for informed consent.

## Results

### Maximum likelihood estimation

Estimates of tumor growth rate and age at symptomatic parameters are presented in Table 2. The results of the maximum likelihood estimation were consistent with the previous study by [33], but lower than the estimates, *a* = *b* = 1.385 and η = 0.0002566 in similar studies [32,35], this may be due to larger invasive tumor, most being grade 3 and other biological factors compared to the previous studies, because our data consisted of late clinically diagnosed tumors. Our results suggest a slower growth rate, which means we expect more tumors to be detected at the screen, and hence the higher proportion of screen-detected cancers than the proportion of [32] and [35].

### Selection of screening sensitivity under different scenarios

The screening attendance and sensitivity scenarios were simulated using the recommended age of 40 to 74 years and a screening frequency of every two years for the average-risk population, as practiced in most developed countries [35] to calibrate appropriate screening population for our study.

According to Table 3, the proportion of cases detected through screening was 38.21%, 43.22%, and 46.50% for low, moderate and high sensitivity, respectively, with perfect screening attendance. These figures were lower than the 35.2% to 53% reported in [35]. Under imperfect screening attendance, the proportions were 30.79%, 35.05%, and 38.67% for low, moderate, and high sensitivity, respectively. These results are consistent with the 30% to 46% range found in [32], but fall below the 27.1% to 42.1% range reported [35].

**Table 3:** Effect of different screening scenarios on screening program

		% of screen detection		
Attendance	Sensitivity	40–$74^{\text{a}}$	40–$74^{\text{b}1}$	30–$65^{\text{b}2}$
Perfect	Low	35.2	38.21	46.19
Perfect	Moderate	45.1	43.22	52.30
Perfect	High	53.0	46.50	56.65
Imperfect	Low	27.1	30.79	36.85
Imperfect	Moderate	35.3	35.05	42.12
Imperfect	High	42.1	38.67	46.45

^a^Previous study in Sweden [35] using a screening age of 40-74 years, ^b1^using a screening age of 40-74 years for Ghana, and ^b2^using screening age of 30-65 years for Ghana. Note: We expect the Ghana proportion to be higher than Sweden’s due to the smaller growth rate parameter.

We anticipated that the detection rates of screen-detected tumors would be greater than those in previous studies due to expected slower growth rates based on our estimates. However, our findings yielded proportions even lower than those in earlier research (see 40-$74^{\text{b}1}$ in Table 3).

We assumed that imperfect screening attendance and moderate screening sensitivity would more accurately reflect the implementation of the new screening program than other assumptions. Using the proportion of screen-detected cancers from [35], we observed a low detection rate in the targeted group aged 40-70. This suggests that a significant number of cases may be occurring before the screening starts at age 40. Therefore, the established screening age range of 40-74 years may not be suitable for the Ghanaian population based on the results in Table 3, raising concern about the early onset risk. To address this, we adjusted the screening ages to find an optimal range. By comparing our proportions to those reported by [35], we found that adopting a screening age of 30-65 years resulted in better performance than the previous 40-70 years range. The proportions of screen-detected cancers ranged from 36% to 46%, higher than what [32] reported for low to high sensitivity and imperfect screening attendance. These findings were more comparable to the 27.1% to 42.1% range, and the 35.2% to 53% range for imperfect and perfect attendance, respectively, reported by [35], but slightly higher as expected. Additionally, the proportion of diagnoses made at screening (including screen-detected and interval cases) increased from over 68% to 72%. This aligns with the hypothesized average proportion of screen- and interval-detected cancers, estimated to be around 70% for countries with existing screening programs [8].

### Evaluation of early screening ages

We expanded the screening age range from 30 to 65 (see Table 4) to examine how different screening ages impact the screening program and identify how to contribute to the chances of early mammography screening in favor of the Ghanaian population. The results, derived from a thorough research process, indicated the number of cases diagnosed increased with an extended screening age range. For example, screening individuals aged 35 to 74 resulted in more diagnoses than screening those aged 35 to 60. The median number of cases diagnosed during the screening was 22317 (Mean: 22251 ± 2433, IQR: 20678-23949), which was more consistent with the number of cases detected in the 30 to 65 age range. In a previous study, Strandberg *et al*. [29] reported an overdiagnosis rate of 1.9% for the 40 to 70 age group and 2.9% for the 40 to 80 age group, which were lower than estimates for the age groups analyzed in this study. Our study, which extended the screening age range from 30 to 65 (see Table 4), involved a careful process of selecting the screening ages. This process was informed by the probability of cancer detection and the potential risks of harm, such as overdiagnosis and interval cancers, even though mortality reduction is the major indicator of screening program effectiveness, our study was limited in this regard due to lack of model specific data. We found that the extended screening age for each targeted group exhibited less overdiagnosis. Overdiagnosis was defined as the excess number of cancers diagnosed through screening compared to those diagnosed without screening (see Eq (25)). Although all the screening age groups evaluated above might be eligible for early screening program, the optimal early screening program targeted the median age range of 30 to 65 years, as it likely presents lower risks than the higher-detection age ranges listed in Table 4 (e.g., 30 to 70 and 25 to 74).

**Table 4:** Evaluation of the impact of early screening ages on screening program outcome.

Ages (yrs)	No screening	Screening	% Change	% O’diag.	% Screen detected	% Interval cases
35–60	16317	17689	8.22	7.76	33.68	22.03
35–65	18253	19737	8.13	7.52	38.40	23.80
35–70	19480	20464	5.05	4.81	40.19	24.43
35–74	20223	21022	3.95	3.80	41.36	24.85
30–60	18925	20749	9.63	8.79	39.00	26.35
30–65	20875	22107	5.90	5.57	42.14	27.53
30–70	22106	23241	5.13	4.88	48.89	28.50
30–74	22841	23737	3.92	3.77	45.89	28.88
25–60	21143	22527	6.42	6.14	41.57	29.38
25–65	23101	24583	6.42	6.03	46.26	31.20
25–70	24311	25297	4.06	3.40	48.07	31.82
25–74	25064	25863	3.19	3.10	49.18	32.28

The screening results indicate that earlier screening ages may benefit the Ghanaian population compared to the 50-80 years recommended for the United States, based on their population characteristics [41], and the 40-74 years suggested for Northern Europe [8]. However, our assertion on early screening strategy opens up more discussion and research on the subject rather than proposing a standalone screening strategy or policy. Particularly, the chosen screening age is informed by median ages that can optimize detection probability while considering the risks associated with interval cancers, without considering the number of deaths averted or the quality of life added. Furthermore, cost-effectiveness analysis is also a standard indicator for selecting screening ages for different populations; however, this was beyond the scope of the current study [42], and it has not been addressed in the study population.

### Evaluation of screening strategies under different screening frequencies or intervals

While the selection of screening intervals considers the proportion of screen-detected cancers, it is primarily guided by the rates of overdiagnosis and interval cancers. These factors are crucial for monitoring screening effectiveness and mortality reduction. Empirical evidence indicates that the proportion of interval cancers in screening programs is approximately one-fourth [43] or around 20–30% [44] of the screened population. Consequently, this study aimed to design a screening program that minimizes interval cancers as much as possible.

The study specifically evaluated various screening strategies, including the current screening population in Sweden (ages 40–74), the IARC-recommended evidence-based eligible population (ages 50–69), its extension (ages 50–74), and an early screening strategy (ages 30–65). In this context, it is important to note that the screened population acts as the intervention group, while the population without screening serves as the control group. According to the results of our simulated screening program, illustrated in Table 5, the proportion of interval cancers (false negatives) was notably high for the triennial screening interval. In contrast, the proportion of overdiagnosis was high for the annual screening interval. However, the biennial screening interval showed a slight balance or trade-off between overdiagnosis and interval cancers across all strategies, thus offering some advantages in terms of benefits and harm compared to the other intervals. Our findings regarding the relationship between screening frequency and interval cancer align with a previous study that an annual screening frequency results in fewer interval-detected cancers than biennial screening [45]. Similarly, previous studies confirm that yearly screening is associated with a higher risk of false positives or overdiagnosis, particularly among older women, compared to biennial screening, which presents a better risk trade-off [46,47].

**Table 5:** Evaluation of screening strategies under different screening intervals.

Screening intervals	No screening	Screening	% change	% O’diag.	% Screen detected	% Interval cases
Annual						
30–65	20867	22504	7.84	7.27	50.13	20.71
40–74	17249	18223	5.65	5.35	42.03	15.39
50–69	9670	10960	13.34	11.77	26.59	7.91
50–74	10592	11555	9.09	8.33	28.08	8.32
Biennial						
30–65	20867	22099	5.90	5.57	42.03	27.54
40–74	17249	18135	5.14	4.89	36.52	20.62
50–69	9670	10680	10.44	9.46	23.19	10.44
50–74	10592	11466	8.25	7.62	25.01	11.11
Triennial						
30–65	20867	21644	3.72	3.59	34.64	33.49
40–74	17249	17942	3.86	4.01	3.86	25.38
50–69	9670	10557	9.17	8.40	20.11	13.13
50–74	10592	11381	7.45	6.93	21.90	13.96

Screening outcomes for 30–65 - potential early screening strategy, 40–74 - the current Sweden screening strategy,eak 50–69 - IARC recommended strategy, and 50–74 - IARC recommendation extension for annual, biennial, and triennial respectively.

Notably, the proportions of interval cancer for the early screening strategy were 20.71%, 27.54%, and 33.49% for annual, biennial, and triennial screening intervals, respectively. These proportions were higher than those for the other three screening strategies. However, the rates of overdiagnosis were lower (7.27%, 5.57%, and 3.59%) in the early screening strategy compared to the others. Nonetheless, the IARC’s recommendations (ages 50–69 and biennial screening) and its extension provided a better balance between benefits and harms than the early screening and Swedish strategies. Although the proportion of interval cancers for both annual and biennial screening across all strategies fell within the expected range of 20–30% of the screened population, biennial screening resulted in fewer cases of overdiagnosis in all instances compared to annual screening. Therefore, although the early screening strategy offered a better detection advantage for the Ghanaian population, the IARC’s recommendations and extended strategy, which advocate for biennial screening, appear more reliable due to their superior balance of harm trade-offs.

In previous studies, the percentage of overdiagnosis reported for the 50–70 years age group was 14%, 10%, and 6% for annual, biennial, and triennial screening intervals, respectively. For the 40–70 years age group, the overdiagnosis rates were 13%, 7%, and 7% for annual, biennial, and triennial screening intervals, respectively [48]. The higher proportion of overdiagnosis associated with shorter screening intervals, such as annual, aligns with our findings.

The study recommended a biennial (every two years) screening interval for the Ghanaian population, citing the benefits of improved detection and reduced overdiagnosis. However, several factors could further influence the choice of screening interval, including specific risk profiles, economic considerations, the costs associated with each screening strategy, and the interests of stakeholders or policy directions [49]. These factors were not addressed in the current study but could be explored in future research, where the insights and expertise of the audience will be crucial.

### Model screening program

The performance of the screening program was evaluated by comparing the number of clinically detected cancers, referred to as interval cancers, against the number of screen-detected cancers. This evaluation also considered the potential for overdiagnosis. An effective screening program should increase the number of cancers detected while reducing the incidence of interval cancers and overdiagnosis. Interval cancers occur between screening intervals when cancer is detected after a negative mammogram but before the next scheduled screening [13]. Overdiagnosis refers to cancers that would not have been detected during an individual’s lifetime without screening, resulting in a cumulative excess of diagnosed cancers at the time of screening. An optimal screening program should also detect a high proportion of smaller tumors (tumor ≤15 mm or ≤20 mm) to facilitate early diagnosis and treatment.

Table 6 presents a descriptive analysis of the simulated optimal screening program. The mean age of the population with no screen-detected cancers was 47 years, slightly lower than the average age of 51 years in our empirical study. The mean age of screen-detected cases was 46 years, indicating that screen-detected tumors are identified about one year earlier than symptomatic cancers. The tumor size distribution for symptomatic detections predominantly fell between 20 mm and 50 mm (58.25%), with a significant proportion measuring over 50 mm (22.19%) and approximately 19.56% classified as small tumors (≤20 mm). In contrast, the size distribution for symptomatic-detected tumors was larger than that for screen-detected tumors, with small tumors accounting for 55.72%, tumors from 20 mm to 50 mm comprising 29.15%, and those measuring over 50 mm making up 15.13%.

**Table 6:** Descriptive summaries of optimal screening program.

Summary	No screening	Screening
Mean age	47	46
First quantile age	39	38
Median age	47	46
Third quantile age	55	54
Number diagnosed	20867	21915
Tumor size 0–9 mm	659 (3.16%)	4403 (20.09%)
Tumor size 10–19 mm	3422 (16.40%)	7808 (35.63%)
Tumor size 20–50 mm	12154 (58.25%)	6388 (29.15%)
Tumor size >50 mm	4632(22.19%)	3315 (15.13%)

According to the Australian screening program outcomes in 2018, 53.3% of screen-detected tumors were small (≤15 mm), compared to 27.6% of symptomatic detections for women aged 50 to 69 during 2002–2012. This screening outcome corroborates our findings and demonstrates that the simulated screening program is robust enough to detect early or small tumors, thereby reducing the incidence of symptomatic cases and preventing breast cancer-related deaths or extending the survival time of cancer patients. The alignment of our simulated screening outcomes with the expected results of effective screening programs further validates our findings. However, the impact of the screening program on survival time was beyond the scope of this study and will be addressed in future research.

The proposed optimal screening program assumes imperfect screening attendance and moderate sensitivity of the screening tests. Screening is recommended for individuals aged 30 to 65 years, with a frequency of every two years. A sensitivity analysis with a variation of ±10% was conducted.

The sensitivity analysis regarding the risk of cancer onset indicated that an increased risk leads to earlier detection of tumors. This also results in a higher number of small tumors being detected than when there is a decreased risk of onset. Similarly, the sensitivity analysis conducted on growth rate parameters (designated as *a* and *b*) revealed that changes in these parameters do not significantly impact the age at which tumors are detected. However, an increased growth rate parameter does enhance the detection of small tumors and vice versa.

Finally, the sensitivity analysis focused on the screening test sensitivity found no significant effect on the age at detection and only minimal impact on tumor size. Increasing the sensitivity of screening tests resulted in a slight increase in the detection of small tumors compared to decreased sensitivity. The limited impact observed in the sensitivity analysis may be attributed to the minor percentage variations applied. However, it’s important to note that a more significant increase in sensitivity could yield significantly better results, as indicated in the screening scenarios discussed above. This underscores the potential for further research and development efforts to improve the effectiveness of cancer screening significantly.

### Properties of the natural history of breast cancer

#### Tumor onset age distribution.

**Fig 1:**
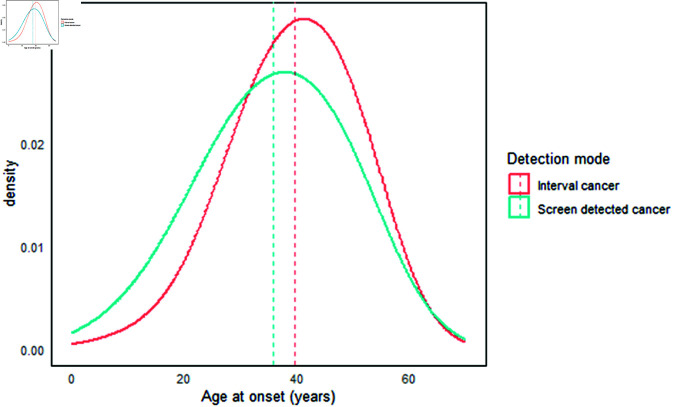
Age at onset distribution. Results reveal a median age of onset of 38 years (mean: 36.57 ± 11, interquartile range: 29–46). Interval cancers appear to onset later, with a median age of 40 years (mean: 40 ± 10, interquartile range: 33–48), compared to screen-detected cases, which have a median age of 37 years (Mean: 36 ± 11 and interquartile range: 28–45).

Screening diagnosis cases include interval (symptomatic cases between scheduled screenings) and screen-detected cases. The age of onset is essential to evaluate a screening program. Fig 1 illustrates the distribution of onset ages for interval cases (in red) and screen-detected cases (in blue) simulated for our screening program.

We used the Moolgavkar-Venzon-Knudson (MVK) two-stage model, as adopted by [8], to simulate tumor onset for the Ghanaian population (West Africa). Our findings reveal a median age of onset of 38 years (mean: 36.57 ± 11, interquartile range (IQR): 29–46). Interval cancers appear to onset later, with a median age of 40 years (mean: 40 ± 10, IQR: 33–48), compared to screen-detected cases, which have a median age of 37 years (Mean: 36 ± 11 and IQR: 28–45). The early age of onset observed in our simulation corroborates earlier findings [50–52].

The distribution of onset age is crucial for determining the appropriate ages for a screening program. Since the onset age can vary across different populations based on their specific risk factors, it is essential to consider both age and cohort for each population [53]. This approach helps determine an effective screening strategy tailored to the needs of that population [54].

#### Tumor size distribution from the simulation.

**Fig 2:**
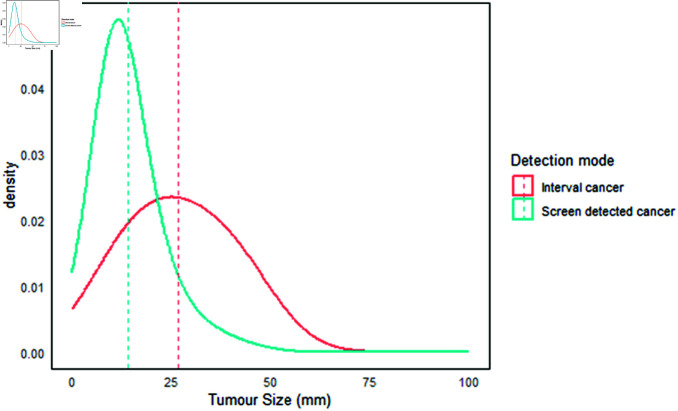
Tumor size distribution. Interval cancers were larger, with a median size of 26 mm (mean = 27 ± 12, interquartile range = 17–37). The median tumor size for screen-detected cases remained smaller at 12 mm (mean = 14 ± 10, interquartile range = 9–18).

**Fig 3:**
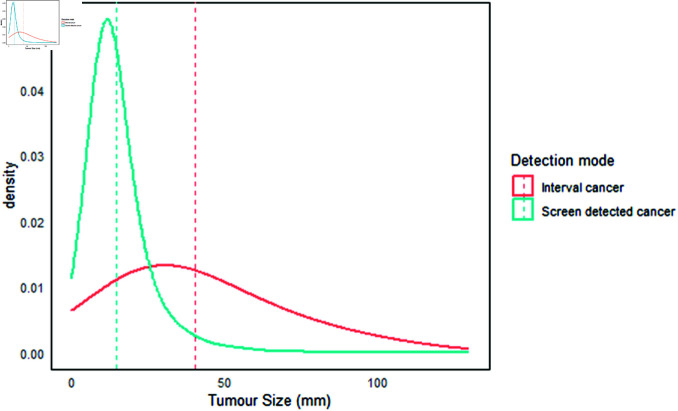
Tumor size distribution. The median tumor size for interval cancers was 35 mm (mean = 41 ± 26, inter quartile range = 21–56). The median tumor size for screen detected cases was 13 mm (mean = 15 ± 10, inter quartile range = 9–18).

The tumor size distribution (tumor diameter) for interval and screen-detected cases, as shown in Figs 2 and 3, indicates that interval cancers generally have a larger tumor size compared to screen-detected cases [55]. When we assumed a maximum tumor size at detection of 120 mm (Fig 3), the median tumor size for interval cancers was 35 mm (mean = 41 ± 26, IQR = 21–56), while the median tumor size for screen-detected cases was 13 mm (mean = 15 ± 10, IQR = 9–18). When we considered a maximum tumor size at detection of ≤50 mm (Fig 2), the results again showed that interval cancers were larger, with a median size of 26 mm (mean = 27 ± 12, IQR = 17–37). In contrast, the median tumor size for screen-detected cases remained smaller at 12 mm (mean = 14 ± 10, IQR = 9–18). In Fig 3, it is evident that most interval cancer cases had tumor sizes between 15 mm and 50 mm, whereas most screen-detected tumors were below 20 mm. These findings highlight the effectiveness of screening programs in detecting smaller tumors, which can enhance treatment outcomes. Smaller tumors tend to respond better to drug concentrations, improving drug efficacy [56,57].

Additionally, tumor size distribution is an important prognostic factor in breast cancer and can assist in planning surgical treatment [58]. Larger tumors (greater than 40 mm) are associated with a poorer prognosis for cancer patients [59]. In comparison, smaller tumors (less than 20 mm) are linked to longer survival lengths and higher survival rates [60].

#### Growth rates to detection mode.

**Fig 4:**
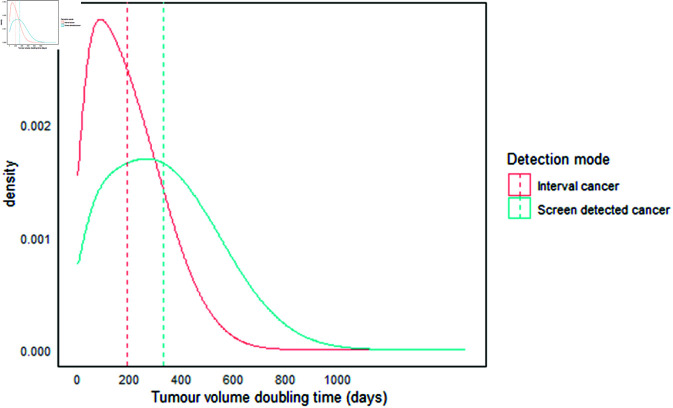
Tumor volume doubling time. The estimated median tumor doubling times are 168 days (mean: 192 ± 137, interquartile range: 81–278) for interval cases and 309 days (mean: 329 ± 208, interquartile range: 162–469) for screen-detected cases.

The tumor inverse growth rate was estimated using the doubling time (DT). We calculated the inverse growth rate distribution for screen-detected and interval cancers based on data from our simulated screening program. The plotted distribution, which illustrates the tumor doubling time in days (i.e., 365 ·ln(2)·r), is presented in Fig 4. Using a simulated large cohort (*n* = 10^6^), the estimated median tumor doubling times were 168 days (mean: 192 ± 137, IQR: 81–278) for interval cases and 309 days (mean: 329 ± 208, IQR: 162–469) for screen-detected cases. Our doubling time estimate for interval cancers is consistent with the 163 days reported by [33]. This result is close to the doubling times of 124 days and 113 days [30], but our results indicate a longer duration compared to the 99 days for interval cases and 170 days for screen-detected tumors reported in [8].

Furthermore, our findings suggest that the average time required for a cancer tumor to grow from 10 mm to 20 mm is over 5 months for interval cancers and approximately 10 months for screen-detected cancers. Thus, our mean DT is shorter than 1.7 years reported [61]. Additionally, [62] found that the mean tumor doubling time for 31 invasive breast cancer tumors was 282 ± 167 days (IQR: 46–794), which is slightly higher than the estimated doubling time for interval cancers in our study.

The estimated tumor doubling time is essential for planning future screening programs, including determining the optimal screening intervals and follow-up times. It also enhances our understanding of breast cancer progression [63,64] and serves as a valuable tool for predicting survival outcomes in breast cancer patients [65]. Additionally, the estimated tumor doubling time provides an evidence-based approach to planning dosing regimens [66,67] for breast cancer patients in Ghana, and it may also aid in evaluating the therapeutic effects of treatment programs [65].

#### Tumor presence time in relation to interval cancers.

**Fig 5:**
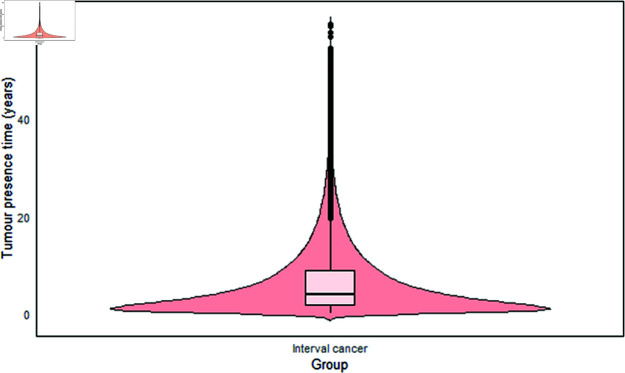
Tumor presence time for interval cancers. The median tumor presence time for interval cancers was 3.9 years, with a mean of 6.46 years (±6.95) and interquartile range of 1.68 to 8.87 years.

In most continuous growth models, tumor presence times are estimated rather than the mean sojourn time, as in Multi-state Markov models. In this study, we successfully estimated the tumor presence time for interval cancers (Fig 5), similar to previous studies [30,38]. Tumor presence time refers to the duration from the onset of the tumor (defined as a tumor size with a diameter of 0.5 mm) to the point at which the tumor becomes clinically detectable or symptomatic. Tumors that proliferate are expected to have a shorter tumor presence time. The median tumor presence time for interval cancers was 3.9 years, with a mean of 6.46 years (± 6.95) and IQR of 1.68 to 8.87 years.

The sojourn time, referred to as tumor presence time in this study, measures the detectable preclinical period of cancer and indicates screening sensitivity or the predicted rate of screen detection [68]. This period serves as a window for early detection; a longer tumor presence time suggests that the tumor is likely detectable by screening for an extended period, whereas a shorter presence time indicates the opposite [69].

In a previous study, the mean sojourn time (MST) was estimated to be 2.3 years (CI: confidence interval, 95% CI: 2.1–2.5) based on a Markov chain model [70]. This estimate was lower than our findings for interval cancers. Another study that estimated sojourn times for different age groups reported mean MSTs of 5.5 years for those aged 50–59 and 6.9 years for those aged 60–69 [61]. Their results were slightly higher than our estimated tumor presence times.

## Discussion

This study examined the natural history of breast cancer in relation to the characteristics of the Ghanaian population, utilizing continuous growth model assumptions to evaluate screening program strategies. We obtained empirical data from the Korle Bu Teaching Hospital and used tumor size to estimate sub-model parameters, including tumor growth rate and age at symptomatic detection. Additionally, we incorporated various assumptions based on GLOBOCAN data [5] regarding age-standardized breast cancer incidence by region to model disease onset and calibrated screening sensitivity from previous studies [32,35] to fit the data from the Ghanaian population. Due to a lack of screening-specific data, previous research [32,38] modeled tumor onset using incidence rates to derive onset risk instead of the MVK carcinogenesis model. In contrast, this study introduced a novel approach under similar circumstances by utilizing the onset model described in [8,38] rather than relying solely on incidence data assumptions. These quantitative efforts allowed us to evaluate potential early screening benefits, which have received less attention due to insufficient evidence from existing and past screening programs, in conjunction with recommendations from international agencies under various screening frequency scenarios.

For early screening in the Ghanaian population, we identified the target age range as 30–65, significantly lower than the recommended starting age supported by existing evidence. Our findings indicated a high detection probability favoring the early screening strategy. We assessed three different screening intervals for our strategies. Our results showed that a higher proportion of interval cancers were associated with triennial and biennial screening compared to annual screenings, which aligns with a previous finding [45]. Our study also found a relatively high proportion of overdiagnosis associated with annual screening compared to biennial and triennial intervals, corroborating earlier research [46,47]. Additionally, biennial screening was identified as providing a better trade-off between interval cancers and overdiagnosis, offering an advantage over both annual and triennial intervals, a conclusion also supported by prior studies [46,47]. Although early screening was associated with more interval cancers, it exhibited a lower proportion of overdiagnosis (likely due to a low growth rate estimate) than other strategies. The IARC’s recommendations for screening starting at ages 50–69 and biennial intervals, as well as the extended guidelines for ages 50–74 and biennial intervals, showed a more satisfactory balance between overdiagnosis and interval cancers, making them more reliable than alternative strategies.

The findings of this study prompt a discussion regarding the benefits of early screening for Ghana (and potentially other African countries), particularly in light of early life expectancy [71] and the rising rate of breast cancer incidence among Ghanaian women under 40 years old [5,72]. However, the effectiveness of any of these screening strategies is primarily linked to their impact on reducing breast cancer mortality and screening sensitivity, interval cancers, and screening standards [9]. Evaluating the effect on breast cancer mortality reduction necessitates reliable empirical data from screening programs, which are currently lacking in Ghana. Future studies with more representative data could extensively demonstrate this, including mortality reduction and estimation of other critical parameters.

Consequently, this study has considered the impact of screening interventions, focusing on monitoring indicators such as inefficiencies and harms—specifically interval cancers and overdiagnosis. This evaluation approach remains relevant for a new screening program until data become available to assess mortality reduction [9]. Furthermore, we have utilized characteristics of the natural history of breast cancer, such as tumor size, growth rate, and duration of tumor presence, to emphasize the importance of early detection through screening while analyzing the effects of these characteristics on breast cancer prognosis and mortality. While studies like these may have some flaws, they have significantly influenced screening program policies in developed countries such as European regions and the United States [9,73,74], which served as a valuable guide for decision-making regarding these strategies. These region-specific studies are important; the current research has evaluated whether mammography screening strategies could be advantageous for Ghana and Africa, mainly as breast cancer incidence and mortality rates are on the rise [3].

The simulated early screening program showed that participants in the screened population detected breast cancer approximately one year earlier than those who were not screened, supporting evidence from countries with established screening programs [32,35]. Moreover, the natural history characteristics revealed a median onset age of 38 years, consistent with a previous study [54]. Additionally, tumors detected through screening were generally smaller and exhibited longer doubling times than those detected in intervals, suggesting a better prognosis for screen-detected cancers.

Despite the overall benefits of the study, there are notable limitations. For instance, the research relied on retrospective data from a patient cohort treated at a single, prominent healthcare facility, where access may be affected by significant socioeconomic factors. The data do not adequately represent the general population, limiting their applicability to the broader Ghanaian context. Additionally, the empirical tumor sizes used for our estimates were derived from invasive and advanced-stage cases, generally larger than those in less advanced situations. This circumstance results in slower growth rates and introduces significant length bias in the study. Furthermore, the evaluation of screening strategies focused solely on detection, harm, and interval cancers, without assessing their impact on mortality reduction—a crucial indicator of effectiveness, even though screening harm also suggests screening effectiveness. The study utilized onset parameters *A*, *B*, and δ, along with sensitivity parameters $\beta_{0}$ and $\beta_{1}$, due to the unavailability of screening-specific data. While these parameters were adopted based on careful assumptions grounded in existing literature and calibration, they may present limitations due to genetic variations between the two regions, which could influence our results. Additionally, a larger sample size could potentially enhance the study’s findings. Further research based on pilot data from opportunistic or centralized mammography in Ghana could provide valuable insights into effective screening strategies and inform national policy. This data can also guide future studies on the cost-effectiveness of screening strategies and the availability of resources.

In conclusion, despite some limitations, this study successfully employed key modeling methods to evaluate screening program strategies that can inform policy decisions. For the first time, we have examined the natural history of breast cancer concerning screening programs in Ghana. The parameters for tumor growth and symptomatic detection were based on empirical data. The study also emphasizes the advantages of incorporating data from other regions to strengthen research in areas where local data may be insufficient while ensuring relevance to the local context. Moreover, this research contributes to the scarcity of literature on screening programs in LMICs and encourages further investigation to facilitate the systematic adoption of mammography in these regions. The findings of this study are crucial for inspiring and guiding future research. Specifically, the results provide crucial insights for developing breast cancer screening programs and related studies to promote early detection, improve survival rates, and reduce screening-related risks, such as interval cancers and overdiagnosis, with a particular focus on early screening strategies. Ultimately, this research advocates for the systematic implementation of mammography in Ghana and other LMICs, aims to enhance breast cancer screening practices and patient treatment plans, and contributes to policy development in Ghana and similar developing nations.

## Other onset functions

Hazard function:

$$h_{T}(t)=\frac{\delta AB(1-e^{(B-A)t})}{Be^{(B-A)t}-A},\;A<0,\;B>0,\;\delta >0.$$ (12)

Survival function is given as:

$$\;G_{T}(t)=P(T>t)={\left[\frac{(B-A)e^{Bt}}{Be^{(B-A)t}-A}\right]}^{\delta },$$ (13)

where:

$$\delta =\tilde{\nu }/\tilde{\alpha },$$ (14)

$$A=\frac{1}{2}[\;(\tilde{\beta }+\tilde{\mu }-\tilde{\alpha })+\sqrt{(\tilde{\beta }+\tilde{\mu }-\tilde{\alpha }{)}^{2}+4\tilde{\alpha }\tilde{\mu }}\;],$$ (15)

$$B=\frac{1}{2}[\;(\tilde{\beta }+\tilde{\mu }-\tilde{\alpha })-\sqrt{(\tilde{\beta }+\tilde{\mu }-\tilde{\alpha }{)}^{2}+4\tilde{\alpha }\tilde{\mu }}\;]$$ (16)

## Maximum likelihood function for parameter estimation

$$L(\theta )=\prod_{i=1}^{n}f_{V_{U}}(\nu_{i})$$ (17)

$$f_{V_{U}}(\nu_{i})=\eta a\frac{b^{a}}{(b+\eta (\nu_{i}-\nu_{o}){)}^{a+1}},\;\nu >\nu_{o}$$ (18)

$$L(\theta )=\prod_{i=1}^{n}\left[\eta a\frac{b^{a}}{(b+\eta (\nu_{i}-\nu_{o}){)}^{a+1}}\right]$$ (19)

$$l(\theta )=\sum_{i=1}^{n}\left[\ln(\eta )+\ln(a)+a\ln(b)+(-(a+1)\ln(b+\eta (\nu_{i}-\nu_{o})))\right]$$ (20)

## A Sub-functions

### A.1 Symptomatic detection

Tumor volume at symptomatic detection at some point in time, in the absence of screening, is given as

$$V(U)=\nu_{o}-\frac{\log(1-u)}{\eta r}$$ (21)

where $\nu_{o}$ is the tumor volume at onset, and *u* is sampled from a uniform distribution. Applying the assumption that tumors are spherical, we can calculate the tumor size or diameter at symptomatic detection as given below

$$d(x)=\sqrt[{3}]{\frac{6}{\pi }V(U)},\;\;\;\;x\geq t,$$ (22)

also, the time at symptomatic detection, U^′^ was given as

$$U^{\prime }=r\ln\left(\frac{V(U)}{\nu_{o}}\right).$$ (23)

### A.2 Screen detection

The diameter of a tumor at age *x* given *R* = *r* and *T* = *t* is given as

$$d_{r,t}(x)=\sqrt[{3}]{\frac{6}{\pi }\nu_{o}e^{\left(x-t\right)/r}},\;\;\;\;x\geq t$$ (24)

The screening program overdiagnosis was also calculated using the expression below.

$$Q=\frac{N_{scr}-N_{abs}}{N_{scr}}$$ (25)

where *Q* is overdiagnosis, *N*_*scr*_ is the number of cases diagnosed at screening, and *N*_*abs*_ is the number of diagnosed cases in the absence of screening.
